# Correlation between Thiopurine S-Methyltransferase Genotype and Adverse Events in Inflammatory Bowel Disease Patients

**DOI:** 10.3390/medicina55080441

**Published:** 2019-08-05

**Authors:** Davide Giuseppe Ribaldone, Alessandro Adriani, Gian Paolo Caviglia, Amedeo De Nicolò, Danilo Agnesod, Marco Simiele, Danila Riganò, Rinaldo Pellicano, Roberto Canaparo, Giovanni Di Perri, Antonio D’Avolio, Francesco Luzza, Ludovico Abenavoli, Giorgio Maria Saracco, Marco Astegiano

**Affiliations:** 1 Department of Medical Sciences, Division of Gastroenterology, University of Torino, 10126 Torino, Italy; 2 Department of General and Specialist Medicine, Gastroenterologia-U, Città della Salute e della Scienza di Torino, C.so Bramante 88, 10126 Turin, Italy; 3 Department of Medical Sciences, Unit of Infectious Diseases, University of Turin, Amedeo di Savoia Hospital, 10149 Turin, Italy; 4 Dipartimento di Scienza e Tecnologia del Farmaco, University of Turin, 10124 Turin, Italy; 5 Department of Health Sciences, University “Magna Graecia”, viale Europa, 88100 Catanzaro, Italy

**Keywords:** Crohn’s disease, inflammatory bowel disease, large intestine, small intestine, ulcerative colitis

## Abstract

*Background and Objectives*: In patients with inflammatory bowel diseases (IBD), the use of azathioprine results in adverse events at a rate of 5% to 20%. The aim of the study was to assess a possible correlation between genetic variability of the enzyme thiopurine S-methyltransferase (TPMT) and the development of toxicity to azathioprine. *Materials and Methods*: A retrospective, single center, blind, case-control study was conducted on 200 IBD patients, of whom 60 cases suspended azathioprine due to toxicity (leukopenia, pancreatitis, hepatitis, and nausea or vomiting), and 140 controls continued treatment with the drug without adverse events. *Results*: In the entire cohort, only 8 cases of heterozygous mutations of TPMT were observed, corresponding to 4% mutated haplotype rate, much lower than that reported in literature (close to 10%). No homozygous mutation was found. Regarding the TPMT allelic variants, we did not find any statistically significant difference between patients who tolerated azathioprine and those who suffered from adverse events. (OR = 0.77, 95% CI = 0.08–7.72; *p* = 0.82). *Conclusions*: According to our study, in IBD patients, the search for TPMT gene mutations before starting treatment with azathioprine is not helpful in predicting the occurrence of adverse events. Importantly, patients with allelic variants should not be denied the therapeutic option of azathioprine, as they may tolerate this drug.

## 1. Introduction

The inflammatory bowel diseases (IBD) include Crohn’s disease (CD) and ulcerative colitis (UC), both characterized by chronic and uncontrolled inflammation of the intestinal mucosa. 

Thiopurines, mainly represented by 6-mercaptopurine and the prodrug azathioprine, are used in cases of steroid-dependent IBD or to reduce the risk of recurrence after surgery in CD [1,2], as they have cytotoxic and immunosuppressive properties [3]. Therefore, 15–28% of patients on treatment with thiopurines have several adverse reactions; mainly gastrointestinal intolerance, and hematological, hepatic, and pancreatic effects [4].

Once inside the cell, azathioprine is converted to 6-mercaptopurine by glutathione-S-transferase and undergoes several metabolic transformations that lead to the production of two different active nucleosides: The conversion of 6-mercaptopurine by the enzyme hypoxanthine guanine phosphoribosyltransferase leads to the formation of 6-thioguanine (6-TG), and the thiopurine methyl-transferase (TPMT) pathway leads to the methylation of 6-mercaptopurine, forming 6-methyl-mercaptopurine riboside (6-MMPr) [3]. These metabolites have some differences in their effect on target cells: The current opinion is that 6-TG may be responsible for the immunosuppressive effect, while 6-MMPr may be a secondary metabolic product (dependent on TPMT activity) with a less effective action, but a stronger toxic effect on the liver [5]. Thus, an overproduction of 6-TG could lead to myelosuppression (due to excessive effect), while an overproduction of 6-MMPr could lead to hepatotoxicity [6]. The activity of TPMT has wide inter-individual variability. Weinshilboum et al. [7], in a randomized study including 298 patients, showed that TPMT activity has a bimodal distribution in the general population: Approximately 89% have high enzymatic activity, 11% intermediate activity, and only 1 out of 300 (0.3%) lack activity. Such variability is partially determined by single nucleotide polymorphisms (SNPs) in the *TPMT* gene [8]. Four allelic variants, called TPMT*2, *3B, *3C and *3A (resulting from the association of mutations *3B and *3C), were found in more than 80% of Caucasian subjects with a middle or low enzymatic activity, of which the most frequent was *3A [9]. Patients with TPMT*3A mutations have a complete loss of enzymatic activity, those with TPMT*3B exhibit a 9-fold reduction in enzyme activity, while in patients with TPMT*3C, enzyme activity falls by 1.4%. In the United States, the Food and Drug Administration has suggested the genetic typing of TPMT before starting azathioprine or 6-mercaptopurine treatment to prevent myelotoxicity [10].

The aim of this study was to evaluate the potential correlation between the genetic variability of TPMT and the development of toxicity associated with azathioprine treatment in patients with IBD.

## 2. Materials and Methods

We included patients with IBD who were followed at the outpatient clinic of the Unit of Gastroenterology, A.O.U. City of Health and Science of Turin, between June 2015 and January 2016. All patients received written information on the study and gave signed informed consent for blood testing and clinical data collection. The study followed the principles of the Declaration of Helsinki and was approved by the local ethical committee (Comitato Etico Interaziendale A.O.U. Città della Salute e della Scienza di Torino—A.O. Ordine Mauriziano—A.S.L. Città di Torino) (approval code 0056825).

In all patients, azathioprine dosage was increased throughout the study (initially 0.5–1 mg/kg daily, then increased over 3–4 weeks to 2.0–2.5 mg/kg daily).

After obtaining written informed consent, patients were divided in two classes: Those who had stopped azathioprine therapy due to toxicity (cases), and those who continued azathioprine treatment (2.0–2.5 [mg/kg]) for at least 4 months without adverse events (controls).

The inclusion criteria were CD or UC diagnosed according to European Crohn’s and Colitis Organization (ECCO) criteria [1,11]. The exclusion criteria were: Start of a new medical therapy for IBD during azathioprine treatment; positive history of hematologic, hepatic, gallbladder or pancreatic diseases; alcohol consumption of more than 20 g/day; and lack of signed informed consent.

Among patients who suspended azathioprine due to toxicity; pancreatitis, hepatitis, leukopenia and gastrointestinal intolerance were reported as side effects. Pancreatitis was defined in the presence of clinical symptoms (abdominal pain which may radiate to the back, often associated with nausea and vomiting, and elevation of serum amylase three times above the upper limit) [12]. Abdominal ultrasound was performed to rule out biliary pancreatitis. Toxic hepatitis was diagnosed in the presence of an elevation of alanine transaminase (ALT) or aspartate aminotransferase (AST) levels ≥2 times the upper limit [13], whereas leukopenia was detected through a white blood cell count of less than 3.0 [× 10^9^/L]. Gastrointestinal intolerance was defined as the new onset of nausea and/or vomiting without abdominal pain and serum alterations of pancreatic/liver enzymes.

A peripheral venous blood sample was taken from all patients to obtain DNA for TPMT genotyping, which was performed blinded to the adverse events. The three most common allelic variants of the *TPMT* gene (TPMT*2, TPMT*3C and TPMT*3B) in the Caucasian population were analyzed. TPMT enzyme genotyping and evaluation of its allelic variants TPMT*2, *3A, *3B, *3C involved: Extraction and quantification of DNA; mutations identification of G238C (TPMT*2), G460A (TPMT*3B), A719G (TPMT*3B); amplification by means of Polymerase Chain Reaction Restriction Fragment Length Polymorphism (PCR-RFLP); and evaluation of allelic variants of the *TPMT* gene.

Based on the genotype of the TPMT enzyme, subjects were later characterized as wild-type homozygotes (*TPMT^W^/TPMT^W^*), heterozygotes (*TPMT^W^*/*TPMT^M^*), or mutated homozygotes (*TPMT^M^*/*TPMT^M^*).

The differences in the allelic frequencies between the respective groups were analyzed by Fisher’s exact test, considering statistical significant as *p* < 0.05. Data were presented as allelic frequencies and percentages in the respective groups. From the contingency tables, calculations of the odds ratio (OR) and confidence interval (CI) at 95% were performed. The statistical analysis was performed by using MedCalc software (version 14.8.1).

## 3. Results

Among the 2427 patients affected by IBD who were followed regularly at our outpatient clinic, 702 were on active treatment or had been previously treated with azathioprine. Of these, 472 were excluded because of concomitant therapy with azathioprine (193), because they had a positive history of hematologic, hepatic, gallbladder, or pancreatic disease (129), or because they had an alcohol consumption of more than 20 g/day (150). Of the remaining 230 patients, 30 refused to participate in the study. 

Thus, 200 patients suffering from IBD were selected, including 60 cases (30%) who had stopped azathioprine therapy due to toxicity, and 140 controls (70%) on stable azathioprine treatment. Of the whole population, 120 (60%) were affected by CD and 80 (40%) by UC. The clinical characteristics of CD patients and UC patients are reported in Table 1.

The most common side effect in patients who had to stop azathioprine was pancreatitis; representing 47% of total adverse events, followed by toxic hepatitis (40% of the total). Leukopenia was found in six cases (10%), and only two cases (3%) of gastrointestinal intolerance were reported (Table 2). 

Among the 60 cases only 2 (3%) showed a heterozygous mutation (*3A). Among the 140 controls, a wild-type (*1/*1) genotype was found in 134 (96%), while 6 (4%) were found to carry a mutation in heterozygosity; 4 (3%) with genotype *3A and 2 (1%) with genotype *2. No mutation on both alleles (homozygous mutation) was observed.

When comparing cases with controls, no statistically significant difference was found. A frequency of allelic variants of 3% (2/60 patients) and 4% (6/140 patients) were detected in cases and controls, respectively (*p* = 0.824). No allelic variant was associated with adverse events related to azathioprine treatment (OR = 0.77, 95% CI = 0.08–7.7; *p* = 0.82). Moreover, no allelic variant was found in patients with leukopenia, hepatitis, or nausea and vomiting.

Considering the pancreatitis side effect alone, no statistically significant difference was found between the frequency of haplotype mutation in patients with pancreatitis compared to those who did not develop it: 7.1% (2/28 patients) and 3.5% (6/172 patients), respectively. No statistically significant correlation was found between *TPMT* gene mutation and azathioprine-induced pancreatitis (OR 2.1, 95% CI 0.2–22; *p* = 0.53). We did not find any statistically significant correlation between *TPMT* gene mutations and gender (4 out of 116 males, 3.4%, 4 out of 84 females, 4.8%; *p* = 1) or IBD type (2 out of 120 CD, 1.7%, 6 out of 80 UC, 7.5%; *p* = 0.3). The differences in the allelic frequencies among the respective groups and relative odds ratios with their 95% CIs are showed in Table 3.

## 4. Discussion

The crucial question as to whether pretreatment TPMT testing performed in IBD patients contributes to decreasing bone marrow suppression-specific mortality induced by azathioprine has long been a matter of debate [14,15]. In a multicenter study, thiopurine therapy was prescribed either according to the classic therapeutic dosage, or the dose adjusted according to the TPMT mutations: The overall proportion of hematological adverse events did not differ between the groups [16].

In our study comprising of 200 patients, a heterozygous mutation was found in only eight patients, corresponding to a 4% frequency of mutated haplotypes. This is significantly lower than the rate reported in the literature (close to 10% [17]), despite the high frequency of adverse events registered in our population. The latter is due to the fact that our study design focused on patients with adverse events. We selected 60 cases that suspended azathioprine due to adverse events. This sample (30% of the study population) is not comparable with the data of any prospective studies that report lower frequencies of adverse events, especially regarding pancreatitis occurrence [18]. Moreover, myelotoxicity often appears after several months of therapy, thus a study period of 4 months could underestimate the myelotoxicity rate, although in the literature leukopenia is reported as an early side effect. 

According to ECCO guidelines [1,2], among the 60 patients with adverse events, azathioprine therapy was stopped. However, it should be noted that in patients with hepatotoxicity, a potential alternative for overcoming intolerance could be treatment with low-dose azathioprine plus allopurinol. In our context, we had to choose the option of stopping treatment due to the patients’ disagreement to continue the same drug.

In six out of eight cases, the *3A heterozygous mutation was found (the most common in Caucasian subjects [3]) while in two out of eight, the *2 heterozygous mutation was found. No homozygous mutation has been found in any subject.

The statistical analysis showed no significant difference between those who tolerated azathioprine and those who had overall adverse events regarding the frequency of the allelic variants of TPMT (OR = 0.77; 95% CI 0.08–7.71; *p* = 0.82). In addition, no statistically significant difference was observed in the rate of haplotype mutation among patients who developed pancreatitis compared to those who did not develop it (7.1%, 2/28 patients, and 3.4%, 6/172 patients, respectively; *p* = 0.53). This is in line with data reported in other studies [19,20]. When considering the type of toxicity, no correlation was found between the TPMT genotype and the development of leukopenia, hepatotoxicity, or nausea and vomiting. Finally, no statistically significant differences were found between the groups obtained by stratification based on gender (*p* = 1) or type of IBD (*p* = 0.3), when comparing the frequency of the allelic variants of TPMT.

A meta-analysis investigated the associations between TPMT polymorphisms and azathioprine-induced adverse events in patients with autoimmune diseases [21]. The results showed that TPMT polymorphisms were significantly associated with azathioprine-induced overall adverse effects, bone marrow toxicity, and gastric intolerance. However, the subgroup analysis according to ethnicity showed a significant association between TPMT polymorphisms and azathioprine-induced bone marrow toxicity in Asian populations, but not in Caucasian populations. The authors concluded that TPMT polymorphisms can explain a variable proportion but not all episodes of azathioprine-related adverse events, and furthermore, a normal TPMT genotype cannot exclude the development of side effects. Thus, TPMT genotyping before starting azathioprine therapy cannot replace the current practice of periodic monitoring of white blood cell count. We agree with the authors’ conclusions and, according to our data, the search for *TPMT* gene mutations before starting azathioprine therapy is not useful to predict adverse events such as leukopenia, pancreatitis, hepatitis, or nausea and vomiting. The exclusion of patients with *TPMT* allelic variants could also deny a therapeutic option to about 4% of those who may tolerate such drug.

Other methods have been studied to detect rare or novel variants of genes accounting for thiopurine toxicity, which are not detectable through standard genotyping, and although a significant association between TPMT activity and some of these variants has been reported, there is no clear advantage over biochemical monitoring for predicting toxicity [22].

A potential limitation of our study is its retrospective nature. However, the potential heterogeneity arising from this is limited by the fact that, in our outpatient clinic, all authors follow International Guidelines [1,2]. Furthermore, during the past 25 years, all consultations have been recorded in both a paper archive and a computerized data bank. Lastly, the case-control design [3] is adequate to find a difference in an objective parameter like a haplotype variant between patients who stopped azathioprine due to an adverse event, and those who tolerated it for a mean time of more than 60 months. Another potential limitation of our study is that, to date, 31 allelic variants (TPMT*2–TPMT*29) are known to be associated with a poor enzymatic activity, compared to the wild-type allele (TPMT*1), and in our study, 4 allelic variants (TPMT*2, *3B, *3C and *3A) were sought. We considered these variants as they are found in more than 80% of Caucasian subjects with middle or missing enzymatic activity [9]. However, this choice could explain the lower frequency of mutated haplotypes detected in our cohort (4%), compared with that reported in the literature (10%) [17]. Furthermore, metabolite levels of 6-TG and 6-MMP were not measured.

## 5. Conclusions

Our results support the ECCO guidelines that do not recommend performing the genetic typing of TPMT before thiopurine therapy is initiated, and suggest close laboratory monitoring, including complete blood count, and pancreatic and hepatic biochemistry.

## Figures and Tables

**Table 1:** The clinical characteristics of the UC patients (n = 80) and CD patients (n = 120).

	**UC Patients**	**CD Patients**
Sex, *n* (%)		
Males	48 (60)	68 (57)
Females	32 (40)	52 (43)
Age at diagnosis (mean age, range)	33 (13–59)	30.5 (16–67)
Azathioprine therapy *n* (%)		
Ongoing	56 (70)	84 (70)
Stopped for adverse events	24 (30)	36 (30)
Average time azathioprine is ongoing (months)	69	59

**Table 2:** Adverse events which led to the stopping of azathioprine therapy, and the TPMT genotype in patients.

Adverse Event, *n* (% among Patients who have Stopped Azathioprine Therapy due to Toxicity)	TPMT Genotype (Number of Patients)
Pancreatitis, 28 (47)	*1/*1 (26)
	*1/*3A (2)
Hepatitis, 24 (40)	*1/*1 (24)
Leukopenia, 6 (10)	*1/*1 (6)
Nausea and vomiting, 2 (3)	*1/*1 (2)

*1/*1 = homozygous wild type genotype; *1/*3A = heterozygous genotype.

**Table 3:** Allelic frequencies among the respective groups and relative odds ratios with their 95% Cis.

	Overall Toxicity ^1^		Pancreatitis ^2^		Gender ^3^		Disease ^4^	
	Yes	No	Yes	No	Males	Females	CD	UC
Mutated	2	6	2	6	4	4	2	6
Wild-Type	58	134	26	166	112	80	118	74
Total	60	140	28	172	116	84	120	80

^1^ OR = 0.77; 95% CI = 0.08–7.7; *p* = 0.82; ^2^ OR = 2.1; 95% CI = 0.2–22; *p* = 0.53; ^3^ OR = 0.72; 95% CI = 0.18–2.98; *p* = 1; ^4^ OR = 0.2; 95% CI = 0.04–1.81; *p* = 0.3.
